# Blaming the young is always more accessible rather than accusing the older employees: an experimental view over age and health in organizations

**DOI:** 10.3389/fpsyg.2024.1340711

**Published:** 2024-06-26

**Authors:** Gabriela-Maria Man, Radu-Ioan Popa, Mihaela Man

**Affiliations:** ^1^Department of Psychology, Faculty of Social Sciences and Humanities, “Lucian Blaga” University of Sibiu, Sibiu, Romania; ^2^Department of Social Work, Journalism, Public Relations and Sociology, Faculty of Social Sciences and Humanities, “Lucian Blaga” University of Sibiu, Sibiu, Romania

**Keywords:** age, health, stereotypes, management strategies, motivation, experiment

## Abstract

**Introduction:**

The stereotype content model postulates that different groups evoke different emotions and reactions based on two dimensions: intention toward others (warmth) and competence.

**Methods:**

In this study, we used an experimental design and a qualitative approach to investigate how managerial strategies are selected and motivated when a subordinate makes a work task related error but belongs to a group that is stereotypical perceived differently in terms of warmth and competence (age groups with or without a medical condition). Thus 75 employees analyzed one of the five hypothetical cases and described the managerial strategy and motivation for usage.

**Results:**

Data revealed that managerial strategies incorporate more active harm elements for younger employees in contrast with vulnerable groups (older employees with unspecified medical conditions, younger or older employees with a medical condition), who benefit from more active facilitation strategies. The strategy usage motivation is also different in the case of younger employees, the control group and the vulnerable groups.

**Discussion:**

The study outcomes bring additional evidence to support the stereotype content model theory and the socioemotional selectivity theory, enriching applicability on organizational practice and human resources management.

## 1 Introduction

In the last decade, the number of older workers has grown in the European Union countries, including Romania (Eurostat, 2023). This change in employment rates based on age has increased diversity within organizations (Grigoli et al., 2018). The diversity phenomenon in work structure and cases also addresses the medical condition of present day workers. European Foundation for the Improvement of Living and Working Conditions (2019) presented that a quarter of the work population in the EU holds a chronic disease, the trend showing an increase in all age groups with a focus on the older employees. From this point of view, cancer as a particular medical condition will mark an estimated 77% increase in the next decades (World Health Organization, 2024).

Diversity within organizations may generate some challenges. The stereotype content model suggests that different social groups (e.g., age groups and adults with medical conditions) can elicit varying reactions based on the stereotypes associated with them (Fiske, 2018). Scholarly literature suggests that age groups and adults with medical conditions are stereotypically depicted differently (Stanciu et al., 2017). This raises the question as to whether managers will act differently when a subordinate makes a work task related error but belongs to a different age or medical condition group.

### 1.1 Age and health as a differentiator in management strategies

In the characterization and categorization of others, age is an often-used criterion (stereotyping).

Younger employees are often depicted as being lazy/unmotivated, unreliable, irresponsible, and selfish (Finkelstein et al., 2013). They are perceived as more likely to make rash decisions, insubordinate (Petery et al., 2020), with poor ethical standards (Raymer et al., 2017), and less warm (less friendly, less kind, less empathetic) (Kleissner and Jahn, 2020; Helaß et al., 2022). In other words, they are stereotypically depicted as less well-intended toward others. However, they are viewed as more favorable in terms of competence dimensions (Cuddy et al., 2008; Fiske, 2018). For instance, they are perceived as capable of performing well, capable of learning, willing to change, and interested in training (Kleissner and Jahn, 2020).

Older employees are often depicted as more well-intended toward others (Kleissner and Jahn, 2020; North, 2022). They are characterized as more loyal, ethical (Petery et al., 2020), responsible, dependable (Vickerstaff and Van der Horst, 2021), trustworthy, supportive (Petery et al., 2020), warmer (friendly, kind, empathetic) (Harris et al., 2018; Kleissner and Jahn, 2020; Vickerstaff and Van der Horst, 2021), being also perceived as leaders (Drydakis et al., 2022). From another point of view, older employees are depicted as less favorable in terms of competence. For instance, they are perceived less trainable (Richardson et al., 2013), slower learners, resistant to change, slow when it comes to completing tasks (Petery et al., 2020), and less competent (Harris et al., 2018; North, 2022).

Adults with different medical conditions or physical limitations may be characterized to a greater extent as being similar to older adults in terms of intention toward others (in terms of warmth) and competence dimensions (Cuddy et al., 2008; Canton et al., 2023). The same patterns were identified in Romania for adults with medical conditions and older adults (Stanciu et al., 2017).

The different characterizations based solely on employees’ ages can be related to different managerial behaviors. In general, age characterization can lead to discrimination for older and younger employees (Chang et al., 2020; Marques et al., 2020; de la Fuente-Núñez et al., 2021; Rothermund et al., 2021). Older employees tend to be discriminated against in terms of recruitment and selection (Neumark et al., 2019; Drydakis et al., 2022; Keskinen et al., 2023; Keskinen and Nikander, 2023), access to training programs or promotion (Previtali et al., 2022), retention strategies (Harris et al., 2018), and so on, underlining behaviors that incorporate avoidance. On the other hand in the case of an error/mistake, the approach may be to ignore the problem if the error is produced by an older employee (Fingerman et al., 2008; Miller et al., 2009). However, errors made by younger employees can elicit more confrontational approaches (active harming behaviors) such as yelling, judging or critiquing, for example (Anderson and Morgan, 2017; Zachariadou et al., 2018).

Studies reveal that employees with medical conditions are checked more frequently by their supervisors upon task performance, even when it is not necessary (Fevre et al., 2013). In addition, employees diagnosed with cancer are seen as needing constant help and special support in performing their tasks (Bae and Cho, 2021), which embodies facilitation approaches. It was pointed out that they tend to be ignored in meetings, discouraged from being involved in tasks and pitied (Knott et al., 2014).

The stereotype content model explains that the characterization intention based toward others (with regard to warmth) and competence (being capable and agentic) generates different patterns of emotions and behaviors toward group members (Fiske, 2018, 2019). Members of groups perceived as being high in warmth but low in competence (such as older adults and adults with medical conditions) evoke pity and sympathy, and elicit helping and protecting behaviors from others (active facilitation behaviors), but also avoidance and neglect (passive harm behaviors). Members of groups who are depicted as being more competent but less warm (such as young adults) evoke envy and jealousy, and elicit more active harming behaviors (critiques, punishment, bullying and so on) but also elicit convenient cooperation for particular tasks, (passive facilitation behaviors) (Cuddy et al., 2008).

Taking all this into consideration, the aim of the present study is to investigate how managerial strategies are selected and motivated when a subordinate makes a work task related error. More specifically, the following research questions are stated:

Q1: How does the employee’s age and medical condition impact the selection of the managerial strategies in case of a work task related error?

Q2: How does the employee’s age and medical condition impact the motivation for choosing a specific managerial strategy?

### 1.2 Future versus present-oriented managers’ strategies

Socioemotional selectivity theory postulates that the time dimension, with time being perceived as limited or open, can influence the activities that people engage in Carstensen (2021). If time is perceived as being limited, people will engage in activities/goals with a shorter time frame, focusing on present activities/goals, as compared to people who perceive time as being open, and who engage in long-term, future orientated activities (Carstensen et al., 2003). Studies reveal that older adults feel that time is more limited than do young adults (Coudin and Lima, 2011; Strough et al., 2016; Liao and Carstensen, 2018). In addition, people with a severe medical condition may perceive time as being more limited (Charles and Carstensen, 1999; Coudin and Lima, 2011; Kooij and Van De Voorde, 2011; Grühn et al., 2016; Zhou et al., 2018). Future time perspective is a variable that is also found in a work context in that employees can perceive that time left in their career is limited (Zacher and Frese, 2009; Topa and Zacher, 2018; Park and Hai, 2021; Alcover et al., 2023), or that prospects or opportunities in their career are limited (Park et al., 2021; Park and Hai, 2021). As expected, older employees perceive career future time as being more limited than do young employees (Zacher and Frese, 2009). In addition, employees who perceive that they have limited career opportunities engage in present activities/professional goals to the detriment of long-term activities which are oriented toward the future (Park et al., 2021). Time perspective is not only an intra-individual assessment. It is also an inter-individual assessment. Time can also be perceived as being open or limited in a specific relationship; for instance, relations with older adults as compared with relations with younger adults can be perceived as more limited (Fingerman et al., 2008). This may be the case also for the relationship with a person with a severe medical condition compared with the relationship with a healthy person. The last goal of this research is also to reveal whether or not managers employ different managerial strategies with regard to employees from different groups who are known to have different time perspectives (old vs. young employees and ill vs. healthy employees); more specific if the strategies are short-term oriented (present) or long-term oriented (future). Taking all this into consideration the study raises a third research question:

Q3: How does the employee’s age and medical condition impact the time frame choices of managerial strategies?

## 2 Materials and methods

### 2.1 Design

The experiment had a two-way design. The related measures for the independent variables were age (with two levels – 29 and 60 years of age) and the medical condition (containing two levels – with medical condition and unspecified medical condition). The dependent variables consisted of management strategies (the respondent had to select three major management strategies they would apply in this role) and motivations (the respondents had to explain each action in terms of arguments used for selecting specific management strategies). Ratings for the dependent variables were made through open-ended questions. The results were approached through a qualitative and in-depth analysis, which goes beyond standard statements of our participants, in order to observe various associations between different patterns of management strategies and motivations, in an organizational setting.

### 2.2 Participants

The present study was conducted in various sectors of activity, in Romania, with participants having an employee status. The eligibility criteria consisted of having a present job at the moment of the experiment. Overall, the sample consisted of 75 participants distributed as follows: 17 in experimental group 1, 16 in experimental group 2, 12 in experimental group 3, 14 in experimental group 4 and 16 in the control group. The age of the overall sample ranged from 18 to 63 years of age (*M* = 40.65, SD = 12.09). Participants’ work experience ranged from 0 to 40 years (*M* = 18.83, SD = 11.475). There were 47 female and 28 male respondents. The Kruskal-Wallis H test showed there were not any significant statistical differences in terms of age scores between the experiment groups, *χ*^2^(4) = 3.757, *p* = 0.440, with a mean rank age score of 39.82 for the experimental group 1, 32.75 for experimental group 2, 34.33 for experimental group 3, 46.79 for experimental group 4 and 36.38 for the control group. Data in the case of work experience did not show any significant statistical differences in scores between the groups, *χ*^2^(4) = 2.916, *p* = 0.572, with a mean rank work experience score of 38.15 for experimental group 1, 33.25 for experimental group 2, 38.75 for experimental group 3, 45.93 for experimental group 4 and 35.09 for the control group. In the case of years of studies, data analysis showed again no significant statistical differences in scores between the groups, *χ*^2^(4) = 5.591, *p* = 0.232 with a mean rank year of studies score of 40.33 for experimental group 1, 40.50 for experimental group 2, 54.42 for experimental group 3, 55.87 for experimental group 4 and 48.56 for the control group. Regarding the participants distribution inside the five study groups, in terms of sex variable, data presented no significant statistical differences *χ*^2^(4) = 1.379, *p* = 0.848 between experimental group 1 (*N* = 17), experimental group 2 (*N* = 16), experimental group 3 (*N* = 12), experimental group 4 (*N* = 14) and control group (*N* = 16) in terms of sex. The sample is a convenience sample. Participants were randomly allocated to the conditions and did not receive any compensation or credit to take part in the experiment.

### 2.3 Procedure

We built a database with employees who met the eligibility criteria, to which we sent email invitations in a progressive way. After being randomly distributed in one of the five study groups, the participants received an email with the invitation to participate in the research. By accessing a Google Form link, the participants received the survey. In the first part of the survey the participants were asked to give an informed consent to take part in the experiment. After giving the informed consent, the participants were invited to read a vignette. Participants who did not answer the invitation email in the first stance, were emailed again one more time only. In total we sent 150 invitations to employees. From these, 92 employees agreed to take part, from which 75 were eligible and were included in the study. We collected the data online. Firstly, each researcher shared his opinion and experience upon age and health topics, secondly the team discussed and analyzed the subjects establishing a common team acceptance framework, thus controlling negative influence bias on data Research Topic and other procedures. We conducted a content analysis, in collaboration with all the team researchers, using a bottom-up approach. First, all the researchers read the answers data in order to prepare the coding process. Each member of the team coded in the first phase 7 participant responses in accordance with the research questions. Secondly we organized several meetings to discuss the resulting codes, observe patterns and common features, building categories and establishing final topics. The process allowed us to build the concept map and the coding scheme. Thirdly, all the researchers analyzed the remaining data and coded them using NVivo qualitative data analysis software, version 11 (QSR International Pty Ltd., released 2015). In order to ensure inter-coder reliability every answer was coded by all 3 members of the research team. By case, differences were discussed, analyzed and solved. Throughout the coding process we updated and refined the concept map and coding scheme, providing topic interpretations and observing associations and interactions. The interrater reliability has ranged from Kappa = 0.78 (*p* < 0.0.001), 95% CI (0.637, 0.892) to 0.98 (p < 0.0.001), 95% CI (0.916, 1).

### 2.4 Vignette

Participants read one of the five different versions of the vignette. After participants completed their ratings, a manipulation check verified that they were aware of the target’s age and medical condition. The five vignettes were as follows: 1. older employee – with medical condition; 2. older employee – unspecified medical condition; 3. younger employee – with medical condition; 4. younger employee – unspecified medical condition; 5. no age and no medical condition specifications (control group). Vignette example model (1): “A 60-year-old person, suffering from cancer, employed as an accountant in a company’s financial department has, among other tasks, to recover the amounts owed by the Health Insurance Company concerning the employees’ medical leave. Due to the non-submission of a refund request, by this person, for the amount owed, within the legal term, the company lost a sum of money. Being asked about the reason for not submitting the refund request of the amounts due, prior to the deadline, the person simply invoked forgetting.”

## 3 Results

**Figure 1:**
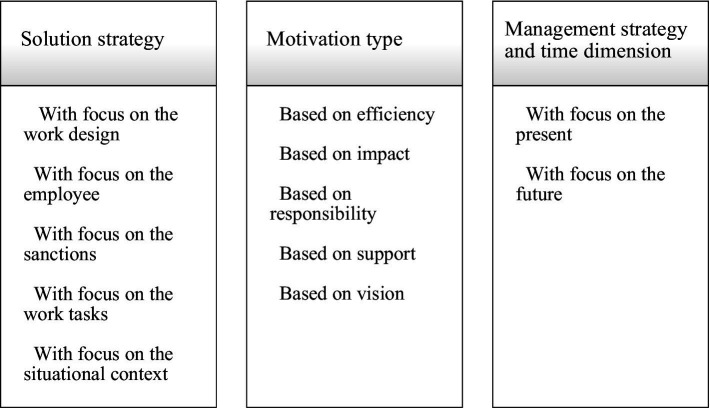
Coding scheme with categories and subcategories data.

Based on the participants’ responses, their experience and perception, and in relation to the five different vignettes, three major categories with distinct subcategories emerged from the data as presented in Figure 1.

### 3.1 Solution strategies

Analyzing and processing participants’ answers from our sample, we managed to structure them into five categories of managerial solution strategies regarding the process of managing the experimental situations.

#### 3.1.1 Solution strategy with focus on the work design

Participants taking the manager role, orient themselves toward department reorganization strategies, work volume recalibration, tasks and employee attributions reevaluation related to job description or job specification and taking actions. Data we obtained states that this strategy is not so frequent inside the experimental conditions. We observed a certain preference, even though in lower levels, mostly in experimental group 1 (older employee – with medical condition) and experimental group 4 (younger employee – unspecified medical condition), where respondents focus more than in other study groups, on taking a work design strategy. At first look, the two study groups seem rather different but the organizational context offers some insight as follows: 1. for the older employee with a medical condition case, the employer seems rather interested in reducing hers or his work volume, easing the job profile and even reorganizing the office or department work where the employee is located; 2. in the case of the younger employee with an unspecified medical condition, the employer connects the work design strategy with a performance and efficiency plan for the future. So if in experimental group 1 the employer focuses on the age and medical variables as key factors on easing the access to work, adapting the job profile to the health and age status of the employee, in experimental group 4, interest falls in supporting the young employees on their professional development for maximum efficiency and performance. Data showed little interest to apply this type of strategy in the case of older employees with unspecified medical condition (experimental group 2) or younger employees with medical condition (experimental group 3). In the case of the control group, where respondents have no information on the age or medical condition of the employee, results showed a certain tendency toward a preference for the use of such strategy.

“…an reevaluation of the work tasks that the person has according to the job description…” (age range 40–45 years) – experimental group 1 (older employee – with medical condition).

“…analyzing the work tasks and work volume of the employee from a performance view…” (age range 50–55 years) – experimental group 4 (younger employee – unspecified medical condition).

“…I would reanalyze the tasks the person needs to fulfill…” (age range 40–45 years) – control group.

#### 3.1.2 Solution strategy with focus on the employee

When regarding the employee directly, respondents in manager role approach the person topic by offering support, organizing discussions to evaluate the employee condition and status, analyzing error impact, offering training options and financial support or appealing to counseling and psychological services. Data we obtained in experimental group 1 (older employee – with medical condition), experimental group 2 (older employee – unspecified medical condition) and experimental group 3 (younger employee – with medical condition), show higher levels. These strategies are frequent in all the experimental conditions, but with a stronger preference in the above mentioned conditions. We may conclude that the medical and age variables condition the support and person oriented policies in organizations, claiming that respondents have a tendency to adopt supportive attitudes toward fellow employees or subordinates when confronted with illness and old age, dimensions which influence these perceptions and behaviors.

“…I would propose that the person should retire based on the health condition, or to get a sick leave at least…” (age range 30–35 years) – experimental group 1 (older employee – with medical condition).

“…checking the person’s health condition from a neurological and psychiatric point of view…” (age range 55–60 years) – experimental group 2 (older employee – unspecified medical condition).

“…to empathize with the ill employee, to offer moral and professional support, to motivate him…” (age range 45–50 years) – experimental group 3 (younger employee – with medical condition).

#### 3.1.3 Solution strategy with focus on the sanctions

In the given vignettes, respondents specified a variety of sanctions they would apply to the employee that caused the work task error, as a solution strategy for the unpleasant organizational situation. A few examples state: financial sanctions, salary cuts, negative evaluation, moving to an inferior job position, job firing, working overtime for retrieving the money etc. In this situation, participants in experimental group 4 (younger employee – unspecified medical condition) and in the control group select mainly strategies focused on sanctions. Data in the other experimental groups (1 – older employee – with medical condition; 2 – older employee – unspecified medical condition and 3 – younger employee – with medical condition) indicated a low level of preference for such actions or strategies. It seems that the age variable becomes more important in this case than the medical condition, determining the managers to be more strict and demanding with younger employees when dealing with a work task related error or negative situation at work. Moreover, participants who do not have any information about the age or the medical condition of the employee tend to choose these types of strategies more often (control group).

“…sanctioning her with position demotion, retrieving the company financial losses from her, making her feel guilty…” (age range 45–50 years) – experimental group 4 (younger employee – unspecified medical condition).

“…moving her to an inferior job position, retrieving the money from her in several months, verbal warning…” (age range 60–65 years) – experimental group 4 (younger employee – unspecified medical condition).

“…I would sanction the person to understand better what he or she has done, I would oblige her to return the money inside the firm one way or another, by various means, to see how difficult it is…” (age range 35–40 years) – control group.

#### 3.1.4 Solution strategy with focus on the work tasks

When it comes to work tasks, respondents become very specific, choosing strategies that reflect punctual activities such as: keeping an Outlook work calendar, monitoring tasks and activities, planning duties and follow up schedule etc. Experimental group 1 (older employee – with medical condition) obtains a certain level of responses but not very high. Data in the case of the other experimental groups (2 – older employee – unspecified medical condition; 3 – younger employee – with medical condition and 4 – younger employee – unspecified medical condition) report low levels of interest on applying such strategies based on work tasks. It would seem that alongside strategies that follow work design changes and employee support for the older employees with medical conditions, task management in detail can be added up as a natural implied process that completes the first two. In the case of the control group, results showed no preference for the use of such a strategy.

“…I would suggest her to keep a daily agenda with the major tasks, e.g., Outlook calendar and checking it frequently…” (age range 55–60 years) – experimental group 1 (older employee – with medical condition).

#### 3.1.5 Solution strategy with focus on the situational context

In terms of situational context, respondents in the manager role choose to apply strategies that would: recover the financial damage in a punctual way, mend the organizational emergency with or without the responsible employee, reveal the real causes for the problem, evaluate short term effects and company damage etc. Data we obtained revealed that experimental group 2 (older employee – unspecified medical condition) involves the higher levels of preference for such strategies. Given the entire script, it would seem that the age variable takes the lead here, explaining that managers reflect more on solution finding and adjustments in the case of an older employee, no matter if there is a medical condition or not. Moreover, it is interesting that when it comes to addressing age and health issues for the employee in a certain given scenario, the manager would focus more on the old, from a situational context point of view. Data in the other experimental groups (1 – older employee – with medical condition; 3 – younger employee – with medical condition and 4 – younger employee – unspecified medical condition) indicated lower levels of preference for such actions or strategies. In the case of the control group, data showed higher interest for managing the situation context when respondents have no information on the age or medical condition of the employee.

“…I would try to find, together with the person in charge, an immediate solution in order to solve the problem that was created…” (age range 35–40 years) – experimental group 2 (older employee – unspecified medical condition).

“…I want to make sure it was a unique faulty situation and I will try to identify the causes that led to this forgetfulness…” (age range 50–55 years) – control group.

### 3.2 Motivation type

Results from the participants’ answers analysis were categorized in five major dimensions given the motivation type used to support and explain managerial strategy selection in the experimental situations.

#### 3.2.1 Motivation based on efficiency

By selecting different motivations for the strategies they would apply, respondents in manager roles, especially in experimental group 4 (younger employee – unspecified medical condition), base their source of motivation on an efficiency model. In other words, respondents take action in organizations, based on efficiency as a major motif when dealing with faulty situations created by young employees. There is also an interesting association with the strategy itself, where the profile would consist of applying more frequent sanctions in the case of younger employees with unspecified medical conditions, motivated by an efficiency claim. The same discourse is used in the case of experimental group 3 but at a lower rate (younger employee – with medical condition), where being efficient triggers and explains the actions and preferred strategies to use, with more focus on work design and employee, highlighting a greater attention toward the health status. It would seem that both conditions indicate that respondents prefer to take action in relation to younger employees, no matter their medical condition, motivating their decisions on an efficiency basis. Data in experimental group 1 (older employee – with medical condition) and experimental group 2 (older employee – unspecified medical condition) indicated lower levels of preference for this motivation type. In the case of the control group, results showed also a certain preference for using this type of motivation, when respondents have no information related to age or medical condition of the employee.

“…remediation of the problem and avoiding such situations in the future…” (age range 25–30 years) – experimental group 4 (younger employee – unspecified medical condition).

“…I would inform the higher management immediately about the incident, and together with a higher authority and the person responsible to find a quick and efficient solution in order to resolve…” (age range 25–30 years) – experimental group 3 (younger employee – with medical condition).

“…first of all it is of utmost importance to quickly resolve the problem the employee created…” (age range 20–25 years) – control group.

#### 3.2.2 Motivation based on impact

Regarding the impact concept, respondents consider the negative toll that the organizational situation might have upon the work quality, performance, department and organization functioning. Data showed that to some low extent, respondents consider the impact view as a motivation when selecting different strategies in experimental group 1 (older employee – with medical condition). In the other experimental groups (2 – older employee – unspecified medical condition, 3 – younger employee – with medical condition and 4 – younger employee – unspecified medical condition), the impact concept reaches lower levels of usage, preference and frequency. Exploring experimental group 1 outcomes, it would seem that when it comes to an older employee with medical condition, managers think more of the impact on the team, office, department or organization work and performance, being affected in a negative manner by the faulty situation generated by the employee, more frequent than in the case of the other experimental groups. In the case of the control group, results indicated some level of preference for this motivation type, in low frequency.

“…in order to reduce the impact on the company and on the employee I am motivated to identify such a solution…” (age range 35–40 years) – experimental group 1 (older employee – with medical condition).

“…it is simple when others cover the losses…” (age range 25–30 years) – control group.

#### 3.2.3 Motivation based on responsibility

In the case of responsibility, respondents choose this type of motivation in order to explain the strategies they take in terms of placing the burden of crisis management either on the manager or on the employee. Data we obtained showed a preference for using this specific motivation especially in experimental group 4 (younger employee – unspecified medical condition), in order to explain the role of the manager to apply sanctions and take further action but also to raise awareness to the employee about its duties. In experimental group 1 (older employee – with medical condition) and experimental group 2 (older employee – unspecified medical condition), data stated with a high percentage the importance of the manager role, placing the view that it is the leader’s responsibility to resolve the matter in which the older employee was involved. The age variable generates a shift from the employee degree of responsibility more to the manager degree of responsibility in this case. In experimental group 3 (younger employee – with medical condition), the responsibility concept reaches lower levels of preference among respondents. In the case of the control group, results showed a higher interest for responsibility as a motivation trigger for taking action when no information is given on the medical condition or the age status of the employee.

“…I consider these [solutions, sanctions, procedures] to be the primary attributions of the manager, for the company’s interest…” (age range 60–65 years) – experimental group 4 (younger employee – unspecified medical condition).

“…I am the manager of that person so I am responsible as well…” (age range 50–55 years) – experimental group 1 (older employee – with medical condition).

“…it is the duty of the entire department and mine as a manager to ensure that all tasks are fulfilled…” (age range 25–30 years) – experimental group 2 (older employee – unspecified medical condition).

“…it is the person responsibility, if she or he has too many things to do it is normal to forget…” (age range 50–55 years) – control group.

#### 3.2.4 Motivation based on support

For the motivation based on support, respondents choose this type of motivation in order to explain their actions in terms of help, supportive attitudes and human understanding for the employee in need. Results showed that support motivation is mainly selected by participants in experimental group 3 (younger employee – with medical condition), followed by experimental group 1 (older employee – with medical condition) and experimental group 2 (older employee – unspecified medical condition). As a supplementary observation, for experimental group 4 (younger employee – unspecified medical condition), none of the respondents have chosen the support concept as a major motivation to explain their strategy or actions to be taken. In the case of the control group, results showed some interest at a lower level for support of employees when no information is given on the medical condition or the age status.

“…the person passes through a difficult phase in which probably needs a time off and also to discuss with the psychologist such health matters…” (age range 25–30 years) – experimental group 3 (younger employee – with medical condition).

“…there are certain situations in which the work capacity of the employees may diminish, errors appear and the employee needs help…” (age range 45–50 years) – experimental group 1 (older employee – with medical condition).

“…I consider it very important to communicate and find out if the person is confronting other problems and if they need help…” (age range 25–30 years) – experimental group 2 (older employee – unspecified medical condition).

#### 3.2.5 Motivation based on vision

In the case of the vision concept, participants in the experiment choose this motivation to explain an action through long term organizational policies, health and work related strategies and management good practices. Data we obtained showed low levels in every experimental group, as a preference for the respondents to choose such a concept in order to motivate their actions or strategies in relation with the employee, no matter old or young, healthy or not. Only in the case of the control group, data showed some level of preference in using the vision perspective when no data is available about the medical condition or age status of the employee.

“…I can learn from this experience for the future…” (age range 30–35 years) – control group.

### 3.3 Management strategy and time dimension

Concerning the management strategy and time dimension, the answers fall into two sub-categories, either focused on the present or on the future, data we obtained underlining the time frame in which the respondents place their strategy choice.

#### 3.3.1 Management strategies focused on the present

Results showed higher levels in experimental group 1 (older employee – with medical condition), experimental group 2 (older employee – unspecified medical condition) and experimental group 3 (younger employee – with medical condition), stating that both age and medical condition variables influence the respondent in the manager role to orient toward strategies with immediate effect and “now” solutions. As a supplementary observation, we witnessed a small difference between the 3 conditions, given by the medical condition variable, in terms of a higher concern and attention given to the older employee and younger employee with a medical condition when compared to the older employee, with no medical condition. Data showed lower levels in the case of experimental group 4 (younger employee – unspecified medical condition). In the case of the control group, data indicated lower levels of preference in focusing on the present when compared to the future.

“…retrieving the lost amounts of money, rediscussing work tasks, organizing personal training…” (age range 50–55 years) – experimental group 1 (older employee – with medical condition).

“…immediate emergency remediation of the situation…” (age range 35–40 years) – experimental group 2 (older employee – unspecified medical condition).

“…reporting the incident immediately to my superior…” (age range 25–30 years) – experimental group 3 (younger employee – with medical condition).

#### 3.3.2 Management strategies focused on the future

Data showed higher levels of preference in experimental group 4 (younger employee – unspecified medical condition), where respondents in the role of manager favor a strategy with regard to the young employee future development and reducing further implications of work task related error. Moreover, the respondents would prefer to apply these types of actions in the case of younger employees with unspecified medical conditions, opposed to the other experimental groups where they would rather select solutions with focus on the present, as presented before. Data showed lower levels in the case of experimental group 1 (older employee – with medical condition), 2 (older employee – unspecified medical condition) and 3 (younger employee – with medical condition). In the case of the control group, data indicated higher levels of preference in using management strategies focused on the future.

“…preventing such situations in the future and taking measures that can tackle them and stop such incidents from repeating…” (age range 60–65 years) – experimental group 4 (younger employee – unspecified medical condition).

“…to send the person to coaching sessions, in order to succeed in organizing better for the future…” (age range 35–40 years) – control group.

## 4 Discussion

The scholarly literature reveals that different age groups lead to various behaviors/reactions in a work context (de la Fuente-Núñez et al., 2021; Keskinen et al., 2023; Keskinen and Nikander, 2023), but also that employees with medical conditions involve different behaviors/reactions (Carr and Namkung, 2021; Sterkens et al., 2023). However, experimental studies that explore the implications of age and medical conditions on coworkers’ behavior and management approaches are rare. In the present study, we analyzed such implications on managerial strategies, their motivation of usage and the time perspective, in a simulated situational work context.

### 4.1 Managerial strategies

The results emphasize that when a younger employee with an unspecified medical condition makes a work task related error, the managerial approach tends to incorporate active harm strategies, mainly sanctions as the first option (e.g., salary cut, negative evaluation, demotion, dismissal, work overtime). This tendency is also visible in the control group. In contrast, managers tend to incorporate active facilitation strategies when the error is produced by an employee who stereotypically belongs to a vulnerable group, in our case older adult with unspecified medical condition or older/younger adults with a medical condition (Bozzaro et al., 2018; Kuran et al., 2020; Langmann, 2023). For these groups, the participants taking the manager role, orient themselves toward strategies with more focus on the employee (e.g., helping the employee overcome the error, discussing with the employee on the ability to fulfill future tasks, offering psychological counseling, proposing financial aid, suggesting a short leave to recover, reducing the workload and so on).

Differences between the three vulnerable groups mentioned above and the young employee with an unspecified medical condition and the control group, emerged even when the same categories of management strategies were selected. For example, a close look at strategies with focus on the situational context for the vulnerable groups, results showed that participants in the manager role assume actively the solving part as their responsibility. On the contrary, in the case of a young employee with an unspecified medical condition and the control group, participants shift the responsibility toward the employee. This also may reflect more active facilitation behaviors directed toward vulnerable groups. These results are in line with the stereotype content model which postulates that young adults may elicit active harm behaviors more often since they are socially perceived as being competent, capable but lacking trustworthiness and are less well-intentioned (cold), whereas older or ill adults may elicit more active helping behavior since they are perceived socially trustworthy, well-intentioned and friendly (warm) but not so agentic and capable (Cuddy et al., 2008; Fiske, 2019). Our results are also in line with other research that found active harm behaviors to be directed toward young co-workers inside a healthcare context (Anderson and Morgan, 2017; Zachariadou et al., 2018) or support and helping behaviors to be used with ill employees (Bae and Cho, 2021).

Results showed that some participants in the manager role questioned the mental capacity of the older employee with an unspecified medical condition. Answers showed that a psychiatric, psychological or neurologic assessment of the mental capacity of the employee in this case is required. These results may be explained by job-related stereotypes with regard to older employees’ mental capacities. Stereotypically, older employees’ mental abilities are more likely to be questioned when compared with other ages (Posthuma and Campion, 2009; Fernández-Ballesteros et al., 2020). It may also be explained by the fact that, stereotypically, older adults are considered more forgetful (Mazerolle et al., 2021). In our experiment scenarios, the employee mentioned that he/she simply forgot to do the task. For the other two vulnerable groups (older employee with medical condition and younger employee with medical condition) psychological counseling was mentioned as a strategy, motivated by the need to offer more support. The scholarly literature mentions psychological counseling as one of the managerial strategies which facilitates the return to work in the case of employees with cancer (Greidanus et al., 2019). Moreover, the study results underlined a subtle tendency, in some managerial roles, that brings to light the passive harm strategy usage preference for the vulnerable groups condition (e.g., suggesting retirement, suggesting job leave due to problems, searching for a more capable employee etc.). This tendency to use both active facilitation and passive harm behaviors is also in line with the stereotype content model (Cuddy et al., 2008). Moreover, in work-related situations, some co-workers or managers do not know how to approach an employee diagnosed with cancer therefore it is more convenient for them to avoid contact (Yarker et al., 2010).

### 4.2 Motivation type

The participants’ responses in the manager role can be better understood by analyzing their motivations for choosing a particular managerial strategy. The participants’ responses were different when comparing vulnerable groups vs. the control and the younger employee with an unspecified medical condition group. The top motivation for choosing managerial strategies for employees with medical conditions (both younger and older) was support motivation. This was also the second choice of preference for the older employee with an unspecified medical condition. They base their strategy on this type of motivation in order to provide help, support, and show human understanding. On the other hand, this motivation type does not apply in the case of a younger employee with an unspecified medical condition. This aspect reinforces the preference for facilitation behaviors toward vulnerable groups at work. When it comes to younger employees with an unspecified medical condition and the control group, respondents motivate their choice of strategy on the need to raise awareness among employees about duties and responsibilities (responsibility motivation). For these two groups, this motivation explains why sanctions are selected as strategies. The results may be explained by the social perception of younger employees as being capable (Kleissner and Jahn, 2020) but also as being lazy, unreliable, irresponsible (Finkelstein et al., 2013), unwilling to work hard, insubordinate (Petery et al., 2020), and with poor ethical standards (Raymer et al., 2017). The responsibility motivation is less frequently used inside vulnerable groups. However, when this type of motivation appears in the case of the older employee with or without medical condition, it refers to the leader’s own responsibility or co-sharing it when managing errors at work. In general, older adults are socially portrayed as being less competent, but trustworthy (North, 2022), and in particular older employees are more likely to be depicted as being ethical, committed to the job, dependable (Petery et al., 2020) and more cautious (McCann and Keaton, 2013). In conclusion, since older employees (with or without medical conditions) are already seen as a responsible social group, raising their duty and responsibilities awareness comes to no avail. Similar differences to those mentioned above were found in the case of efficiency motivation. This was mainly selected for strategies concerning younger employees. For the younger employee with an unspecified medical condition case it explained sanctions, whereas for the younger employee with a medical condition it described the actions that highlight the need for health status attention. For the first situation, the scholarly literature portrays younger employees as a capable social group (Kleissner and Jahn, 2020; North, 2022). In addition, the efficiency motivation might be also explained by the time perspective, with regard to future oriented strategies, where young employees might be seen as a potential resource for the time to come inside the organization.

### 4.3 Future versus present-oriented managers’ strategies

According to socioemotional selectivity theory, future time perspective changes how goals and actions are prioritized with reference to present-focused versus future-focused at individual level (Carstensen, 2021; Chu and Carstensen, 2023), as well as in the case of employee relationship (Fingerman et al., 2008). At the workplace, employees will often have the tendency to perceive job actions and tasks in the same temporal key note, focusing on the outcome with regard to present or the future, in association with various variables such as impact, expectations, value, costs or degree of engagement. Adopting a managerial role, with regard to a specific strategy, solicits the orientation toward the “now” or the “later” according to the feature of the task. Variables such as employee profile in terms of age and medical condition can be considered as supplementary indicators. Time is perceived as being limited, typically by older adults (Carstensen, 2021) or adults with a medical condition (Zhou et al., 2018), while present-focused actions and goals are prioritized at an intra-individual level (Carstensen, 2021). Following these views, the present study results revealed that management strategies are present-focused for employees that are older, with a medical condition or both, as well as for younger employees with a medical condition, when compared to the control or younger employees with unspecified medical condition groups. This situation confirms the idea that a person detaining a managerial responsibility will likely adopt or prefer a present focused strategy in solving a work related task error when the employee who is responsible belongs to older age category, has a medical condition or the two variables combined. This type of action will be maintained also in the case of young age employees with medical condition. In other words, old age and medical condition define the older and younger employees in terms of present and immediate effect management strategies, focusing on the “now” and rapid solutions creation. Moreover, research data showed a preference for using future-focused strategies in the case of younger employees with unspecified medical condition and the control group. The results may be explained on one hand by the differences in participants’ perception on the time left in a particular professional relationship with an employee from a manager stance (inter-individual time). On the other hand, in the workplace environment, having a manager role implies also a greater attention to vision, long term solutions, problem solving with lasting effect and good practices building and implementation. Solving a young employee faulty task, where no medical condition is present, requires not only attention to an immediate solution but rather a perspective for “later,” consolidating a future strategy for error prevention, guidelines for managing such events and developing a frame for employee training and instruction.

### 4.4 Practical implications

Researching age and health topics should be a high priority due to recent demographic changes, such as the demographic pyramid inversion (Schwarz et al., 2014). In light of current challenges, it is important to develop human resources practices and policies that support older employees and those with medical conditions, so they can extend their careers. Integrating older adults into organizations, as well as adults with medical conditions, must follow through the aging process red lines and trends, active aging policies, return to work procedures and vulnerable groups integration strategies (Popa et al., 2020; Popa and Popa, 2020; Akgüç, 2021). Similarly, it is necessary to establish adaptative human resources practices and policies for younger employees and those without medical conditions in order to retain them inside the organizations for longer periods of time, thus creating appropriate ways of responding to their work-related behavior. This research provides a framework for developing non-discriminatory practices that can be adapted to the unique needs of each employee category. These results can also be used to support the development of social policies that aim to capitalize on existing human resources, ensuring an inclusive climate at the workplace. In these conditions, it is necessary, from a human resources policy perspective, to extend the non-discrimination approach that promotes every employee at the workplace no matter the age or medical category they belong to United Nations (2002). Understanding the particular way of managing age or medical conditions in organizations provides material for appropriate training programs development with regard to the diversity theme, raising awareness and building a healthy work environment. It is important for managers and employees alike to understand, apply and promote a fundamental diversity management related to age and medical variables, avoiding negative discrimination against young and healthy employees while positively discriminating against the vulnerable groups, in relation to task related errors. Last but not least, the present study secures a path toward a yet not fully explored area which associates age, health and stereotyping in the organizational context, enriching the scholarly literature with regard to an extended debate and comparison on the matter.

### 4.5 Limits and future research

One of the research constraints might be related to the limited possibility in anticipating the overlap degree between the participant’s answer in real job-related settings and the answer provided for vignettes in an experimental situation. There might be a potential difference between adults perception on their probable behavior and the real conduct when facing real life situations (Hennink and Kaiser, 2022). Even so, responses in experimental settings still generate ways of conduct and problem solving strategies which can offer insight on the stances the participants play with regard to promoted variables and situational designs. It should be noted that not all individuals who were invited to participate in the research chose to respond, which may limit the findings of the study and affect its external validity. Implications for external validity arise especially when the profiles of those who did not respond to the invitation for study participation differ substantially from those of the participants (Stone-Romero, 2004) or due to the usage of vignettes for which the external validity is not yet fully documented (Eifler and Petzold, 2019; Tremblay et al., 2022).

To gain a better understanding of how managers handle task-related errors, considering the age and medical condition of their employees, it would be beneficial for future research to investigate how managers’ age impacts their choice of strategies and related motivations. It would be useful to analyze not only the managers’ chronological age but also their subjective and biological age in this context. For example, subjective age can be taken into account, as it can become a more accurate criterion for aging, compared to chronological age (Hughes and Touron, 2021). These views are also justified by research insights which state that adults of similar chronological ages have different levels of health status (Crimmins et al., 2021) and different biological ages. Therefore, studying subjective age and biological age is practically useful, as people’s evaluations are often influenced by categories that are perceived to be similar to their own (Riordan and Wayne, 2008).

It is commonly understood that employees are often perceived as more suitable for certain positions and tasks based on their demographic features (Turek and Henkens, 2020). Therefore, future research should focus on investigating task-related errors for different positions requiring different types of tasks. For instance, some tasks may require physical skills while others may involve more decision-making abilities.

### 4.6 Conclusion

When a younger employee with an unspecified medical condition makes a work task related error, the managerial approach tends to incorporate more active harm strategies (sanctions). The selection of such a strategy is mainly motivated by the need to raise awareness on the employees’ duties or responsibilities and to promote efficiency. In addition, managerial strategies tend to be more future-focused. When the same work task related error is made by an employee from a vulnerable group, the approach tends to incorporate more active facilitation strategies (employee and context focus). These are mainly motivated by the need to provide support or to emphasize the co-shared responsibility when managing errors at work. With regard to the vulnerable groups, the managerial strategies are more present-focused. These conclusions mainly support the postulates of the stereotype content model (Cuddy et al., 2008) and of socioemotional selectivity theory (Carstensen, 2021).

## Data availability statement

The datasets presented in this article are not available to the public due to privacy reasons. Requests concerning the datasets should be directed to gabriela.man@ulbsibiu.ro.

## Ethics statement

The study was approved by “Lucian Blaga” University of Sibiu Ethics Review Board (Approval nr. 7/05.08.2020). The study was conducted in accordance with the local legislation and institutional requirements. Written informed consent to participate in this study was provided by each participant.

## Author contributions

G-MM: Writing – review & editing, Writing – original draft. R-IP: Writing – review & editing, Writing – original draft. MM: Writing – review & editing, Writing – original draft.
